# Clarifying the Actual Situation of Old-Old Adults with Unknown Health Conditions and Those Indifferent to Health Using the National Health Insurance Database (KDB) System

**DOI:** 10.3390/geriatrics9060156

**Published:** 2024-12-06

**Authors:** Mio Kitamura, Takaharu Goto, Tetsuo Ichikawa, Yasuhiko Shirayama

**Affiliations:** 1 Department of Community Medical and Welfare, Tokushima University Graduate School of Biomedical Sciences, Tokushima 770-8504, Japan; kitamura.mio@tokushima-u.ac.jp; 2 Department of Prosthodontics and Oral Rehabilitation, Tokushima University Graduate School of Biomedical Sciences, Tokushima 770-8504, Japan; tak510@tokushima-u.ac.jp; 3 Tokushima University, Tokushima 770-8504, Japan; ichi@tokushima-u.ac.jp

**Keywords:** the National Health Insurance Database (KDB) system, individuals with unknown health conditions, individuals indifferent to health, Kayoinoba user, old-old adults

## Abstract

**Background/Objectives**: This study aimed to investigate the actual situation of individuals with unknown health conditions (UHCs) and those indifferent to health (IH) among old-old adults (OOAs) aged 75 years and above using the National Health Insurance Database (KDB) system. **Methods**: A total of 102 individuals with no history of medical examinations were selected from the KDB system in a city in Japan. Data were collected through home visit interviews and blood pressure monitors distributed by public health nurses (PHNs) from Community Comprehensive Support Centers (CCSCs). The collected data included personal attributes, health concern levels, and responses to a 15-item OOA questionnaire. Semi-structured interviews were conducted with seven PHNs. The control group consisted of 76 users of the “Kayoinoba” service (Kayoinoba users: KUs). **Results**: Of the 83 individuals who could be interviewed, 50 (49.0%) were classified as UHCs and 11 (10.8%) were classified as IH, including 5 from the low health concern group and 6 who refused to participate. In the word cloud generated from the PHNs’ interviews, the words and phrases “community welfare commissioner”, “community development”, “blood pressure monitor”, “troublesome”, “suspicious”, and “young” were highlighted. In the comparison of health assessments between UHCs and KUs, “body weight loss” and “cognitive function” were more prevalent among KUs, and “smoking” and “social participation” were more prevalent among UHCs. **Conclusions**: The home visit activities of CCSCs utilizing the KDB system may contribute to an understanding of the actual situation of UHCs, including IHs, among OOAs. UHCs (including patients with IH status) had a higher proportion of risk factors related to smoking and lower social participation than KUs.

## 1. Introduction

As the proportion of individuals aged 75 years or older (old-old adults [OOAs]) in Japan continues to increase, improving the efficiency of medical and long-term care services has become crucial. Efficient care involves the optimal use of limited resources to enhance the health and longevity of the population [1]. Accordingly, large-scale health databases are being developed worldwide [2,3]. In Denmark and other Nordic countries, public institutions manage personal information including national health data, and register-based statistical surveys have been established [4]. In the Netherlands, health data are centrally managed by Dutch Hospital Data (DHD). There is also a data center established by health insurance companies, which enables the secondary use of health data outside the statistical office [5]. Japan’s National Health Insurance Database (Kokuho Database—KDB) system began operations in 2013, following the establishment of the medical care system for OOAs in 2008 [6]. The KDB system enables municipalities to centrally manage medical, dental, and health checkups and long-term care visits for those insured under “National health insurance”, “Long-term care insurance”, and “Medical care systems for late-stage older adults”. Currently, the KDB system is used for intervention research, targeting insured individuals with lifestyle-related illnesses. However, private enterprises and research institutions still do not have direct access to the data [7,8].

The KDB system has enabled the identification of individuals with limited or no access to medical and long-term care who may be in a state of latent vulnerability within the population. Ishida et al. demonstrated that individuals with unknown health conditions (UHCs) had a significantly higher risk of developing moderate-to-severe functional disability or facing mortality within 2 years than those who had undergone medical or health checkups [9]. UHCs are defined as “individuals for whom ‘medical or dental examinations,’ ‘health checkups,’ or ‘long-term care certification’ could not be confirmed by the insurer (municipality) for a certain period” [10]. Additionally, some UHCs are indifferent to their own health (IH), showing no concern for the maintenance of their health. Ozawa et al. reported that health concern factors include “health awareness”, “health motivation”, and “health values” [11]. Fukuda et al. tentatively defined IHs as “people with low health concern and at risk for health decline”, but there is still no clear definition, and efficient detection methods have not been standardized [12]. Fukuda et al. also argued that health interventions targeting IHs, which fall outside both population-based and high-risk approaches, are crucial [13,14,15]. Although there is a clear need to understand the actual situation and to implement interventions, to the best of our knowledge, no study has directly addressed the occurrence of UHCs and IHs among older adults or clarified their health status.

In this study, we operationally defined UHCs as “individuals for whom ‘medical or dental examinations,’ ‘health checkups,’ or ‘long-term care certification’ could not be confirmed by the KDB system for a certain period” and IHs as “individuals with low health concerns among the UHCs or those who refuse health interventions”. The aim of this study was to clarify the actual numbers and health status of UHCs and IHs among OOAs by utilizing the KDB system and the activities of Community Comprehensive Support Centers (CCSCs).

## 2. Materials and Methods

This study employed a cross-sectional design utilizing three surveys: the home visit interview survey, the Kayoinoba survey, and the interview survey of public health nurses (PHNs).

### 2.1. Home Visit Interview Survey

The database used for this survey was the KDB system. A total of 12,053 OOAs were selected as the target population in City A, located in the southern region of Tokushima Prefecture (total population: 69,824; aging rate: 33.9%; as of January 2023). The selection criteria for the survey were as follows: individuals who became OOAs by 31 March 2021 and had no record of “medical or dental examinations”, “health checkups”, or “long-term care certification” during the approximately 4-year period from 1 April 2019 to the date of selection (22 July 2022). On the basis of these criteria, 107 OOAs were selected.

The survey participants were distributed among the six CCSCs in City A in an effort to allocate approximately the same number of individuals to each center. As a result, 102 (51 males and 51 females; mean age—80.1 ± 5.1 years) OOAs were finally selected for the survey.

PHNs from CCSCs personally visited the survey participants. To encourage voluntary participation, the PHNs distributed health equipment (blood pressure monitors) to participants in exchange for cooperation. This approach applied the “nudge theory”, which involves gently encouraging and guiding individuals toward desired behaviors [16]. In preparation, the principal investigator, a social worker, and seven PHNs collaborated to ensure consistency in the survey content and assessment methods.

The survey items included personal attributes (sex and age), health concern level, and 15 questions specifically for OOAs. Health concern levels were measured on a 5-point scale (1 = not interested to 5 = very interested), with those selecting 1 or 2 classified under the low health concern group. Table 1 shows the questionnaire items included in the medical checkups for OOAs. The questionnaire was designed to comprehensively assess the health status of OOAs, considering characteristics such as frailty, and was intended for use at health checkups and places (“Kayoinoba”) where older adults gather [17,18].

The 15 questions were divided into 10 categories: “health condition”, “mental health”, “dietary behavior”, “oral function”, “body weight loss”, “physical function and falls”, “cognitive function”, “smoking”, “social participation”, and “social support”. In this study, based on the guidelines of the Japan Geriatrics Society and the studies by Hori et al. and Nagano et al., negative responses to each item were identified as indicative of health risks [19,20,21]. Individuals under each category were classified as at risk if they provided one or more responses indicating a health risk.

The survey period lasted from 11 October to 30 November 2022.

### 2.2. Kayoinoba Survey

The survey participants were OOAs, designated as Kayoinoba Users (KUs), who were regularly using the six Kayoinoba and who were already receiving health interventions from CCSCs, representing the control group for UHCs. Kayoinoba refers to community health activities promoted by Japan’s Ministry of Health, Labour, and Welfare (MHLW) aimed at preventing long-term care needs and extending healthy life expectancy [22]. Participants with incomplete survey responses were excluded from the analysis.

The principal investigator and a social worker (a 28-year-old) visited six Kayoinoba and conducted interviews with the participants. The survey items were the same as those used in the home visit interview surveys. The survey period lasted from 10 October to 10 November 2023.

### 2.3. Interview Survey of PHNs

Seven PHNs from the CCSCs who had participated in the home visit interview survey were interviewed by a senior PHN (72-year-old) and social worker. The interviews followed a semi-structured format, with each session lasting approximately 1 h. The survey period was from 1 December 2022 to 10 January 2023.

The interview topics included the following: “What you noticed during the home visit interview survey”, “Challenges faced during the survey”, “Positive aspects of being involved in this study”, and “How the CCSCs should evolve in the future to enhance the health awareness of local residents”.

The contents of the interviews with the seven PHNs were quantitatively analyzed using verbatim transcripts. This study utilized text mining software (User Local, Inc., Tokyo, Japan) to generate a word cloud [23]. The analysis employed the term frequency–inverse document frequency (TF-IDF) method, which represents the importance of words by their size. Word extraction was limited to nouns and adjectives, with a maximum limit of 30 words. To better highlight the characteristics of UHCs, place names, personal names, numbers, “CCSC”, and “PHN” were excluded from the analysis. The word cloud was analyzed in Japanese and the results of this analysis are described in English in this paper.

### 2.4. Statistical Analysis

For continuous variables, the Shapiro–Wilk test was performed, and when normality could not be confirmed, comparisons were made using the Mann–Whitney U test. Sex distribution and the proportions of individuals at risk in each questionnaire category were compared between the two groups using a chi-square test. Statistical analysis was performed using the Statistical Package for the Social Sciences (SPSS^®^) software (version 28.0; IBM Corp., Armonk, NY, USA), with a significance level of 5%.

### 2.5. Ethical Considerations

The home visit interview survey was conducted in collaboration with City A as part of a project started in 2022 by the MHLW (Project for Health Promotion and Health Improvement for Older Adults; Theme No. 78, Mizuho Research & Technologies, Inc., Tokyo, Japan). The data were provided after all personal information was anonymized by City A. Therefore, the data used in this study were considered secondary data.

The Kayoinoba survey and the interview surveys of PHNs were approved by the Ethics Committee for Research on Life Science and Medical Care at Tokushima University Hospital (approval number: 4230). The principal investigator explained the details of the study to all participants and obtained their consent before conducting the study.

## 3. Results

### 3.1. Home Visit Interview Survey and Kayoinoba Surve

**Figure 1:**
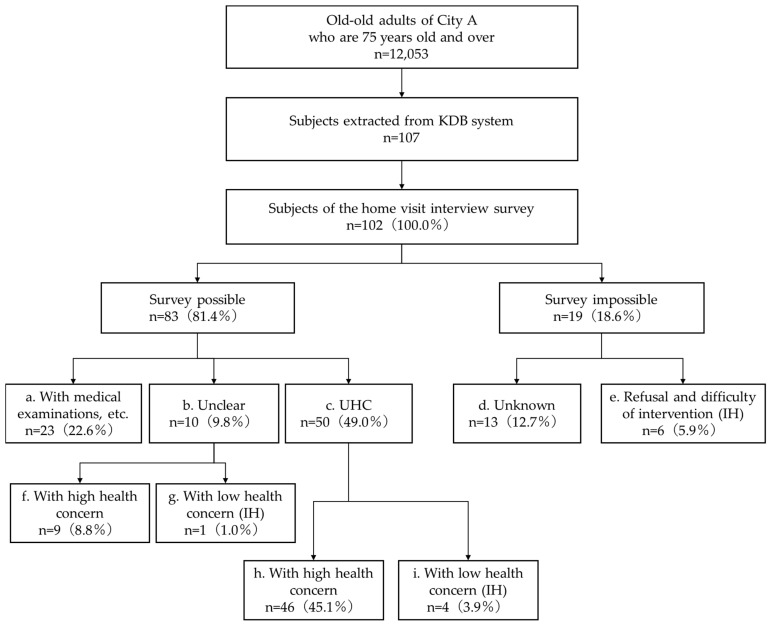
Flowchart and results of the home visit interview survey. a: Individuals who received medical examinations. b: Individuals whose receipt of medical examinations was unclear. c: Individuals with unknown health conditions (UHCs). d: Individuals who were classified as “unknown” due to missing data, absence from their residence, or institutionalization. e: Individuals who refused intervention and for whom intervention was deemed difficult (categorized as IH). f: Individuals with high health concern whose receipt of medical examinations was unclear. g: Individuals with low health concern whose receipt of medical examinations was unclear (categorized as IH). h: UHCs with high health concern. i: UHCs with low health concern (categorized as IH).

Figure 1 shows the selection flowchart and results of the home visit interview survey. Of the 102 participants, 83 (81.4%) were successfully surveyed and provided responses. Of these, 23 (22.6%) had already undergone medical examinations at the time of the survey, 10 (9.8%) were unsure about undergoing medical examinations, and 50 (49.0%) were defined as UHCs. Of the 50 UHCs, 4 (3.9%) were in the low health concern group. Of the 10 participants who were unclear about receiving medical examinations, 1 (1.0%) was in the low health concern group. Of the 19 participants (18.6%) who could not be surveyed and did not provide responses, 13 (12.7%) were classified as “unknown” due to missing data, absence from their residence, or institutionalization. Six participants (5.9%) declined to participate in the survey and were deemed ineligible for intervention by the PHNs. Based on the above, the IH group defined in this study consisted of 4 participants with low health concern out of the 50 UHCs, 1 with low health concern out of the 10 participants who were unclear about receiving medical examinations, and 6 participants who declined to participate in the survey and were deemed ineligible for intervention by the PHNs, totaling 11 (10.8%).

Among the 81 KUs in the Kayoinoba survey, 76 respondents had no missing values, of whom 2 were in the low health concern group. The five individuals with missing data were excluded from the analysis.

Table 2 compares the personal attributes and health conditions of the UHC group (50 participants) and the KU group (76 participants). No statistically significant differences in age were observed between groups (*p* = 0.158). The sex distribution in the UHC and KU groups was 25 males (50.0%) and 25 females (50.0%), and 18 males (23.7%) and 58 females (76.3%), respectively. The proportion of women in the KU group was significantly higher than that of men (*p* < 0.01). Health concern level was significantly lower among UHCs compared to KUs (*p* < 0.01).

On comparing the risk factors by health assessment category between the UHC and KU groups, we found that “body weight loss” was more prevalent in the KU group, at 14.5% (11 participants), than in the UHC group, at 2.0% (1 participant) (*p* = 0.020). “Cognitive function” was more prevalent in the KU group, at 35.5% (27 participants), than in the UHC group, at 18.0% (9 participants) (*p* = 0.033). “Smoking” was more prevalent in the UHC group, at 22.0% (11 participants), than in the KU group, at 1.3% (1 participant) (*p* < 0.01). “Social participation” was more prevalent in the UHC group, at 26.0% (13 participants), than in the KU group, at 5.3% (4 participants) (*p* < 0.01). A post-hoc power analysis was conducted using G*Power 3.1.9.6 (University of Kiel, Kiel, Germany) with an effect size of 0.85, an α value of 0.05, and sample sizes of 50 for UHC and 76 for KU. The result indicated a high power of 0.99.

### 3.2. Interview Survey of PHNs

**Figure 2:**
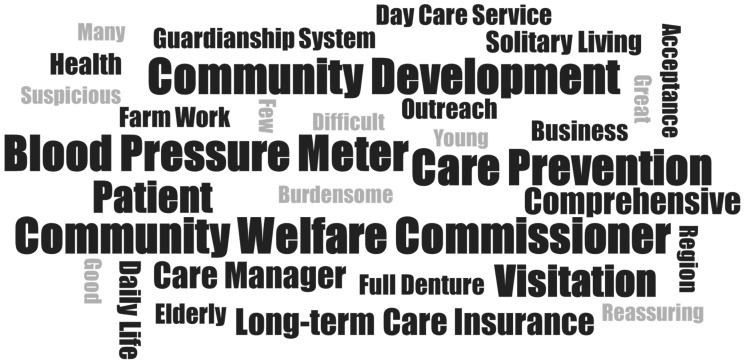
The word cloud generated from the interview survey of public health nurses.

Figure 2 shows the word cloud generated from the interviews with PHNs. As a result of analyzing the transcripts of the interviews with 7 participants, “community welfare commissioner”, “community development”, and “blood pressure monitor”, were the most commonly represented nouns. “Troublesome”, “suspicious”, and “young”, were the most commonly represented adjectives. Regarding the characteristics of UHCs, they were described as lively and “young”, they engaged in “farm work”, and they gave the impression of being “troublesome” and “suspicious”. One respondent commented that UHCs had long been pointed out as being at risk of “solitary living” and social isolation, and this study provided an opportunity to visit them. The effect of the “blood pressure monitor” was that it enabled health guidance to be provided on site, and those who tended to refuse were more likely to “accept” the survey. One respondent commented that it is necessary for CCSCs to collaborate with a “community welfare commissioner”, municipalities, and others to promote “community development”.

## 4. Discussion

The target area, City A, had a population of 69,824 and an aging rate of 33.9% as of 2023, similar to the average population per municipality in Japan (72,130 people) [24] and showing no significant difference compared to Japan’s overall aging rate of 29.0% [25]. When the number of OOAs living in City A (12,053) was used as the base population, the rates of UHCs and IHs were 0.41% and 0.09%, respectively. If these percentages were applied to Japan’s population of people aged 75 years and over (19.37 million), the estimated numbers of those with UHC and IH status would be approximately 80,000 and 17,000, respectively.

In 2020, the MHLW added “Integrated implementation of health services and preventive care for the older adults” to the roles of CCSCs [26]. This initiative involves the collaboration of CCSCs with public health departments to assess the health of older adults. In line with this project, to our knowledge, this study is the first to clarify the actual situation of people with UHC and IH status through cooperation between public health departments and CCSCs in Japan. The findings revealed that individuals in the UHC group had a higher risk of smoking and lower levels of social participation than those in the KU group. Globally, smoking is the leading cause of lung cancer and has been addressed through various medical interventions [27,28]. Although some UHCs have no significant health issues, many of them still smoke. Murakami et al. reported that the risk of death from smoking was particularly high among older men [29,30]. Therefore, smoking cessation guidance is a critical component of health interventions for UHCs.

Regarding the issue of social participation, Fujita et al. and Sakurai et al. reported a high likelihood of the reduction in the frequency of going outdoors translating into physical and cognitive impairments in the future [31,32]. Furthermore, when the risk associated with social participation is viewed as “social isolation”, those in a state of social isolation have been reported to experience a decline in higher-order life functions after 4 years and a reduction in survival rates after 6 years [33]. In contrast, many individuals in the KU group face issues, such as “body weight loss” and “cognitive decline”, which may conversely serve as evidence that Kayoinoba is effectively providing frail individuals with opportunities for social engagement [34,35]. Therefore, it is essential for CCSCs, such as Kayoinoba, to actively guide UHCs toward community resources that offer opportunities for social participation.

In addition, all 4 participants with IH status in the UHC group were male, and 8 of the 11 participants with IH status overall were male. Our study included more female KUs. Previous studies have shown that men tend to be less interested in health than women, and the findings of our study regarding OOAs were consistent with those of the previous studies [36,37]. Furthermore, a lack of interest in health is often a characteristic of middle-aged and older men, whereas women, especially older women, tend to have a wider range of support networks and closer relationships than older men [38,39,40], suggesting that health interventions targeting men, not only in their later years but also starting in earlier stages, will likely be necessary.

Analysis of the interviews with PHNs suggests that they provided valuable insights into facilitating easier access to UHCs and IHs. This study employed a method based on nudge theory, using the distribution of blood pressure monitors as an incentive for survey participation. PHNs reported that “some UHCs and IHs initially displayed rejecting attitudes during visits, describing the interaction as ‘suspicious’ or ‘troublesome’”. However, they also noted that “the act of handing over blood pressure monitors and explaining their usage deepened communication, eventually leading to the smooth ‘acceptance’ of the survey”. Consequently, the application of nudge theory was confirmed to be effective in engaging UHCs and IHs. On the other hand, 5.9% of individuals either declined to participate in the survey or were deemed ineligible for intervention by the PHNs. It is therefore necessary to explore additional approaches that not only involve providing tangible items but also address and alleviate negative psychological traits toward health interventions among UHCs in the future.

This study had three limitations. The first limitation is that as this was a cross-sectional study, it revealed the current situation and health risks of UHCs, including IHs, but it remains unclear to what extent smoking and social participation affect the health of UHCs. Therefore, it is necessary to investigate the longitudinal changes in the health risks of UHCs over time in future studies.

The second limitation concerns the sampling bias of the participants. The research is limited to City A, and it is also estimated that there may be bias in the control group (KUs), making it difficult to generalize immediately. However, this attempt at estimating the numbers of UHCs and IHs using the KDB system is highly meaningful and has great potential in contributing to future health activities in CCSCs.

The third limitation is the barrier to using health data under the current system owing to privacy protection concerns. While this study was approved by the MHLW as a “Project for Health Promotion and Health Improvement for Older Adults”, and the necessary data were provided, it is still challenging to share health data with CCSCs on a daily basis. Anonymization in statistical surveys, along with laws and guidelines for personal information protection, is necessary to promote the use of health data in the future.

In light of the above, it is essential to clarify the causal relationship between the health risks of UHCs/IHs and various factors by expanding the survey area, comparing it with a wide range of control groups, and conducting follow-up surveys. It is also important to further explore the potential of utilizing the KDB system and to consider more efficient health intervention methods for UHCs/IHs.

## 5. Conclusions

The home visit activities of CCSCs utilizing the KDB system may contribute to an understanding of the actual situation of UHCs, including IHs, among OOAs. The UHC group, including those with IH status, had a higher proportion of people at risk owing to smoking and low social participation than the KU group.

## Figures and Tables

**Table 1:** The questionnaire for medical checkup of old-old adults.

Category	Item	At Risk = 1; No Risk = 0
Health condition	1. How is your health condition?	Excellent, good, fair = 0; poor, very poor = 1
Mental health	2. Are you satisfied with your daily life?	Satisfied, moderately satisfied, fair = 0; moderately dissatisfied, dissatisfied = 1
Dietary behavior	3. Do you eat three times a day?	Yes = 0; no = 1
Oral function	4. Do you have any difficulties eating tough foods compared to 6 months ago?	No = 0; yes = 1
	5. Have you choked on your tea or soup recently?	No = 0; yes = 1
Body weight loss	6. Have you lost 2 kg or more in the past 6 months?	No = 0; yes = 1
Physical function and falls	7. Do you think you walk slower than before?	No = 0; yes = 1
	8. Have you experienced a fall in the past year?	No = 0; yes = 1
	9. Do you go for a walk for your health at least once a week?	Yes = 0; no = 1
Cognitive function	10. Do your family or friends point out your memory loss? (e.g., “You ask the same question over and over again”.)	No = 0; yes = 1
	11. Do you find yourself not knowing today’s date?	No = 0; yes = 1
Smoking	12. Do you smoke?	Yes = 1; no, quit = 0
Social participation	13. Do you go out at least once a week?	Yes = 0; no = 1
	14. Do you keep regular communication with your family and friends?	Yes = 0; no = 1
Social support	15. When you are not feeling well, do you have anyone you can talk with?	Yes = 0; no = 1

**Table 2:** Comparisons of personal attributes and health conditions between the groups of individuals with unknown health conditions (UHCs) and Kayoinoba users (KUs).

	UHC (*n* = 50)		KU (*n* = 76)		*p*-Value
**Age (years)** †	79	(75–92)	80	(75–93)	0.158
**Sex (persons)** ‡					
Male	25	(50.0)	18	(23.7)	<0.01 **
Female	25	(50.0)	58	(76.3)	
**Health concern level (1~5)** †	3	(1–5)	4	(2–5)	<0.01 **
**At risk by health assessment category (persons)** ‡					
Health condition	2	(4.0)	11	(14.5)	0.059
Mental health	1	(2.0)	5	(6.6)	0.238
Dietary behavior	5	(10.0)	3	(3.9)	0.173
Oral function	20	(40.0)	33	(43.4)	0.704
Body weight loss	1	(2.0)	11	(14.5)	0.020 *
Physical function and falls	32	(64.0)	53	(69.7)	0.501
Cognitive function	9	(18.0)	27	(35.5)	0.033 *
Smoking	11	(22.0)	1	(1.3)	<0.01 **
Social participation	13	(26.0)	4	(5.3)	<0.01 **
Social support	1	(2.0)	0	(0.0)	0.216

* p < 0.05, ** *p* < 0.01. † Median (Min–Max) with the Mann–Whitney U-test. ‡ Number (%) with Pearson’s chi-square test. Numbers in parentheses indicate percentages in each category.

## Data Availability

The data that support the findings of this study are available from the corresponding author upon reasonable request.
